# Bracket Failure in Orthodontic Patients: The Incidence and the Influence of Different Factors

**DOI:** 10.1155/2022/5128870

**Published:** 2022-01-11

**Authors:** Haris Khan, Samer Mheissen, Ayesha Iqbal, Ali Raza Jafri, Mohammad Khursheed Alam

**Affiliations:** ^1^CMH Institute of Dentistry Lahore, National University of Medical Sciences, Punjab, Pakistan; ^2^Orthodontic Department, Syrian Ministry of Health Private Practice, Damascus, Syrian Arab Republic, Syria; ^3^IOD CMH, Lahore, Pakistan; ^4^Akhtar Saeed Medical and Dental College & Consultant Orthodontist Ittefaq Hospital Trust & Saadan Hospital Lahore, Pakistan; ^5^Orthodontic Division, College of Dentistry, Jouf University, Saudi Arabia

## Abstract

Failure of brackets is a common problem in orthodontics. This affects the treatment time, cost, and compliance of the patient. This study was conducted to estimate the bracket failure rate and the related factors for the long term. *Methodology.* This ambidirectional cohort study included 150 nonsyndromic orthodontic patients undergoing fixed appliance therapy for the last two years. The same patients were followed for 7 months. Different variables related to bracket failure were evaluated. The available data were analyzed descriptively, and the Kaplan-Meier estimate was used to measure the bracket survival rate from the date of bonding to failure. *Results*. A total of 180 bracket bond failures in the 150 included patients (52.2% males and 47.8% females) with a median age of 17 years (range 10-25 years). 69% of brackets failures were reported within the first 6 months after bonding. About 58.3% of bracket failure was noticed in adolescent patients before the age of 18 years. The majority of the cohort (81.1%) has good oral hygiene. The failure rate in patients with normal overbite was 41.1%, in decreased overbite cases was 15%, while in deep bite cases the failure rate was 43.9% with a statistically significant difference. Adults show less bracket failure (41.7%) than adolescent patients (58.3%). More bracket failure was noted in the lower arch (55%) than the upper arch (45%), and there were more bond failures posteriorly (61%) than on the anterior teeth (39%). Majority (41.1%) of the bracket failed on round NiTi wires. *Conclusion*. The bracket failure rate was 6.4%, with most bracket failure occurring in the first 6 months after bonding with individual difference. There was more incidence of bond failure in an increased overbite, adolescents, lower arch, posterior teeth, and lighter alignment wires.

## 1. Introduction

Orthodontics encompasses treatment modalities to correct dentoalveolar malocclusions aiming to restore dentofacial esthetics and function. The most accepted method to achieve these goals is the use of fixed appliances. In fixed appliance orthodontics, brackets are bonded to apply forces from archwires and other auxiliaries to the dentition. The edgewise brackets were introduced in 1928 by Edward Angle [1]. Traditionally, these brackets were welded to orthodontic bands, and then, bands were cemented on the teeth [2], but Buonocore [3] in 1955 introduced acid etching which paved the path for attaching brackets directly to the teeth. Later, Newman et al. [4] pioneered the idea of bonding brackets with composite resin on the surfaces of treated enamel, which is now a standard method of bonding.

One of the inevitable problems encountered in fixed orthodontics is bond failure. In good clinical practice, the failure of brackets should not exceed more than 6% [5]. But an incidence of 0.6-28.3% has been reported in a systematic review [6]. A bracket rebonded due to failure can increase the treatment duration from 0.3 to 0.6 months [7, 8]. Bukhari et al. reported [9] that for every 6 months increase in treatment time, patient compliance to follow their appointments decreased by 23%. Thus, the cost of treatment is enormously increased both for orthodontic practice and for the patient [5].

Multiple patient and operator-related factors affect the incidence of bond failure. Patient-related factors include preexisting enamel or dentine defects, age [10, 11], compliance to treatment [10], oral hygiene, jaw (maxilla or mandible) [12], anterior or posterior teeth [8], overbite [12], and overjet [12]. Operator-related factors like the pattern of etching, etchant concentration [13], type of primer [5], type of composite resin [14], type of curing lamps [15], curing time, bracket material [8, 12], and bleaching procedure carried out before orthodontic treatment [16] can affect the bracket failure rate.

Numerous studies have been done to sort out different factors associated with bracket failure during orthodontic treatment. Most of these studies were retrospective [8, 11], having a small sample size [17], or were followed for a short term [5, 8, 12]. This study was done to investigate the bracket failure rate and the related factors for the long term by ambidirectional design.

## 2. Material and Method

The sample included patients undergoing fixed orthodontic treatment for the last two years in a single orthodontic center. The inclusion criteria comprised nonsyndromic patients having full records and were bonded by the same adhesive (Transbond™ XT, 3 M Unitek, Monrovia, CA, USA) and metallic brackets. All these brackets were from the same manufacturer and bonded using the same protocol. The same patients were followed from December 2018 to June 2019. Data were retrieved from patients' files for any previous bracket failure incidence, and also, the new incidence of failed brackets was noted. The following details were recorded for each patient:

1. Biographical details (name, age, and gender)
2. Time of bracket failure
3. Tooth number
4. Type of wire
5. Overbite configurations (normal 30-50%, increased > 50%, and decreased < 30%) [18]
6. Patient oral hygiene

Patients with clefts, syndromes, or brackets debonded by the clinician, brackets bonded by a different adhesive or protocol, and brackets from a different company were excluded from the study.

Five operators with at least 2 years of experience bonded all the brackets (Lancer® MBT Rx) using the same following protocol: (a) cleaning with pumice, (b) etching with 37% phosphoric acid for 15 seconds, (c) bonding with 3 M™ Transbond™ XT primer and adhesive, (d) curing with Mectron® light-curing system with standard specifications on each use (wavelength: 440-465 nm; intensity: 1.400 mW/cm^2^; and time: 20 seconds).

The overall bracket failure rate was assessed for the whole sample. The data regarding the bracket failure rate was associated with the enlisted variables.

### 2.1. Statistical Analysis

Descriptive statistics were reported for the findings. Shapiro-Wilk test was used to check the normality of the data. As the data was not normally distributed, nonparametric tests (Mann–Whitney and Kruskal-Wallis) were used to investigate the analytical statistics. For the survival analysis, Kaplan-Meier estimate was used. Chi-square and Fisher exact tests were used for categorical data. All statistical analyses were conducted using Stata 15.1 (Stata Corp, Texas, USA) and R Software version 4.1.2 (R Foundation for Statistical Computing, Vienna, Austria).

## 3. Results

**Figure 1:**
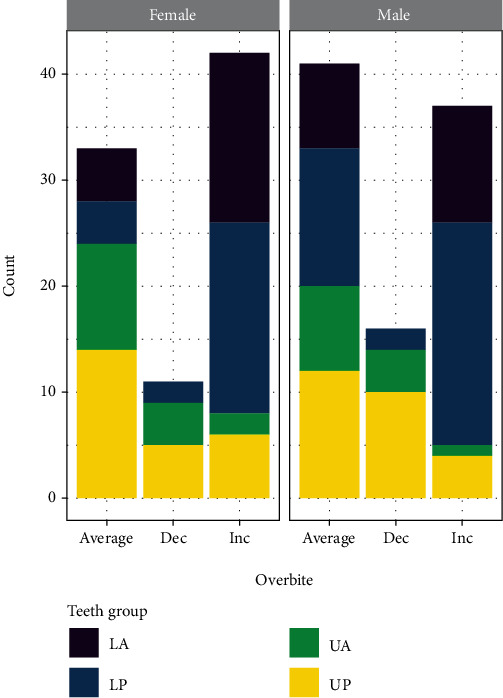
The bracket failures rate in different genders and the effect of the overbite on it. LA: lower anterior teeth; LP: lower posterior teeth; UA: upper anterior teeth; UP: upper posterior teeth; dec: decreased; inc: increased.

150 out of 280 patients undergoing orthodontic treatment were included in the study. 62 patients were excluded due to insufficient records, 56 patients have bonded brackets from a different manufacturer or bonded by a different adhesive or protocol, and 12 patients had clefts/syndromes. There was a first-time incidence of 180 bracket bond failures in 52.2% males and 47.8% females with a median age of 17 years (range 10-25 years) (Figure 1). The bracket failure rate was 6.4% for the first time and 0.5% for the second (nine brackets) and third time (six brackets) failure. The second and third bracket failure was most common on the lower second premolar. Most of the patients, 81.1%, had good oral hygiene, while 13.9% had average oral hygiene. Only 5% of patients have poor oral hygiene.

**Figure 2:**
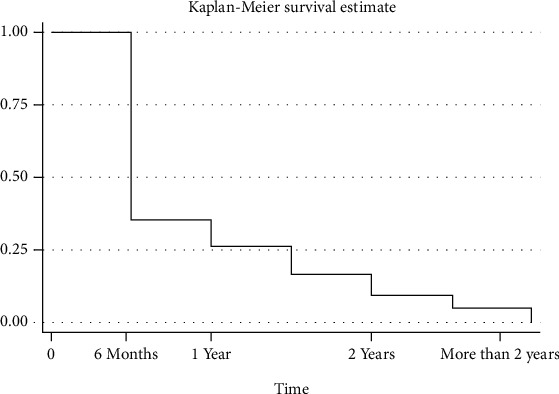
Bracket failure rate over time.

The frequency of the bracket failure is presented in Table 1. The majority of bracket bond failures 69%, were within the first 6 months after bonding. (Figure 2).

About 58.3% of brackets failure was noticed in adolescent patients before the age of 18 years. Most of the bracket failure occurs on the left lower second premolar (12.2%) followed by right lower second premolars (9.4%), while the upper left central incisor reported has the least frequency (1.1%) of bracket failure (Table 2). Bond failure was more common on posterior teeth (61%), especially on lower posteriors (33.3%) (Table 1).

The failure rate in patients with normal overbite was 41.1%, in decreased overbite cases was 15%, while in deep bite cases, the failure rate was 43.9% with a statistically significant difference. The lower posterior teeth have most of the failures in case of an increased overbite, while in case of decreased overbite, most bond failures were noticed on upper posterior teeth (Table 1).

About 41.1% (74/180) of brackets failed on round NiTi wires (0.016^″^ NiTi). 30% (54/180) brackets failed on rectangular NiTi wires, while only (28.9%) of the brackets failed on 0.019 × 0.025^″^ stainless steel (SS) wire.

## 4. Discussion

Most of the studies related to bracket survival rate measured only one-time bracket failure on the same tooth [19–21]. This is done to eliminate the effect of clustering of data. However, Papageorgiou and Pandis [11] proposed that such reporting will underestimate the total failure rate. The bracket failure rate was 6.4% for the first time and less than 0.5% for the second and third times in the present study. Similar bracket failure rates (6-8%) were reported in other studies during the whole course of orthodontic treatment [7, 19]. However, some studies [17, 22] have reported lower bracket failure rates. This can be due to the difference in inclusion criteria and study design of these studies from the present study. Both of the previous studies were prospective with the patient having good oral hygiene. Also, these studies [17, 22] either excluded extraction cases [17] or only used balanced extraction [22] cases while measuring the bracket failure rate.

Nearly two-third (69%) of the bracket failure happened in the first six months after boding. This finding is consistent with the clinical findings of other studies [10, 11, 23]. With regard to the failure frequency, the highest frequency in the recent study was seen in one patient who reported seven incidences of different bracket failure. Less than one-third (30%) of patients reported a single incidence of bracket failure. This is in agreement with previous studies that reported two-third of the patients to have an incidence of multiple bracket failures while only one-third of the patients reported a single incidence of failure [7, 19].

Considering the age factor, almost 58.3% of the bracket failure occurred in adolescent patients (<18 years old). Various reasons could lead to a higher failure incidence in the younger population, such as thick gingival biotype, trauma, and habits [24]. Similar findings were reported by Barbosa et al. [10], who suggested that internal motivation is one of the reasons for better cooperation and a low rate of bracket failure in adult patients. In the present study, males had a slightly higher incidence (52.2%) of bracket failure than females (47.8%), but that was not statistically significant. Similar findings were reported by a randomized clinical trial [25]. However, the literature has controversial findings on this aspect of brackets failure [19, 26–28].

Most of the bracket failures (61%) were reported on posterior teeth, especially the lower second premolars. These results agree with previous studies that reported more bracket failures on posterior teeth than anterior teeth and more failure on lower second premolars [11, 17, 29]. The failure rate of the posterior bracket (premolars) was two times higher than the anterior brackets (incisors and canine). A comparative clinical trial by Mavropoulos et al. [30] reported posterior bracket failure three times higher than anterior brackets. Regarding the failure rate between the lower and upper jaws, our findings were similar to other studies [12, 19, 31]. The higher incidence of bracket failure rate was in the mandibular dental arch compared to the maxillary dental arch. This could be due to the more effect of masticatory forces in the lower arch, impact from the upper teeth cusps, and poor bonding due to inadequate moisture control.

Deep bite cases reported more bracket failures than average or open bite cases with a statistically significant difference. Similar findings were reported by Atashi and Shahamfar [32] in their epidemiologic survey. Most bracket failures in deep bite cases were noticed on lower premolars, so adding bite blocks in these cases can decrease the incidence of bond failure. There was no significant effect of skeletal relationship on bond failure rates which is in agreement with previous studies [33]. In terms of the association between wire and bracket failure, most failed brackets were reported on the (0.016^″^ NiTi) wire. This finding is quite logical as in our study and other studies, as discussed before, most failures occur in the first six months of treatment when the leveling and alignment are taking place on round NiTi wires.

Apart from regional and demographic differences, the settings of this study were similar to the real-world setting as the authors comprised all the orthodontic patients in the department. Interestingly, the confounding in orthodontic studies is common, as there are still slight differences between orthodontists regarding bonding procedures, light-curing tools, isolation, and practitioner's experience.

## 5. Conclusion

In this study, the bracket failure rate was 6.4%, with most bracket failure occurring in the first 6 months after bonding with an individual difference. Adult patients have less bracket failure than adolescent patients with more failure in the lower arch. Also, increased overbite was associated with an increased bracket failure rate.

## Figures and Tables

**Table 1:** Association between bracket failure and different variables.

Teeth group		Mandible *N* (percentage)		Maxilla *N* (percentage)		*P* value
		Anterior	Posterior	Anterior	Posterior	
Overbite	Average overbite	13 (7.2)	17 (9.4)	18 (10)	26 (14.4)	**<0.01**
	Decreased overbite	0	4 (2.2)	8 (4.4)	15 (8.3)	
	Increased overbite	27 (15)	39 (21.7)	3 (1.7)	10 (5.56)	

Age group	<18	19 (10.6)	33 (18.3)	21 (11.7)	32 (17.8)	0.17
	≥18	21 (11.7)	27 (15)	8 (4.4)	19 (10.6)	

Gender	Female	21 (11.7)	24 (13.3)	16 (8.9)	25 (13.9)	0.47
	Male	19 (10.6)	36 (20)	13 (7.2)	26 (14.4)	

Skeletal class	Class I	7 (3.9)	12 (6.7)	8 (4.4)	12 (6.7)	0.09
	Class II	29 (16.1)	44 (24.4)	19 (10.6)	27 (15)	
	Class III	4 (2.2)	4 (2.2)	2 (1.1)	12 (6.7)	

Period	Less than 6 months	31 (17.2)	41 (22.78)	25 (13.9)	28 (15.6)	**<0.01**
	6-11 months	2 (1.1)	8 (4.4)	0	5 (2.8)	
	12-23 months	2 (1.1)	5 (2.8)	4 (2.2)	14 (7.8)	
	More than 24 months	5 (2.8)	6 (3.3)	0	4 (2.2)	

Wire	16 NiTi	20 (11.1)	30 (16.7)	8 (4.4)	16 (8.9)	0.07
	16∗22 NiTi	7 (3.9)	6 (3.3)	10 (5.6)	12 (6.7)	
	19∗25 NiTi	3 (1.7)	4 (2.2)	5 (2.8)	7 (3.9)	
	19∗25 SS	10 (5.6)	20 (11.1)	6 (3.3)	16 (8.9)	

Total		40 (22.2)	60 (33.3)	29 (16.1)	51 (28)	

**Table 2:** Frequency of bracket failure on individual teeth.

Tooth number (FDI)	Frequency	Percent
11	4	2.2
12	7	3.9
13	6	3.3
14	14	7.8
15	13	7.2
21	2	1.1
22	6	3.3
23	4	2.2
24	9	5.0
25	12	6.7
31	7	3.9
32	8	4.4
33	7	3.9
34	9	5.0
35	22	12.2
41	6	3.3
42	10	5.6
43	4	2.2
44	13	7.2
45	17	9.4
Total	180	100.0

## Data Availability

Data is available on request.

## References

[1] Angle E. H. (1932). *Orthodontic appliance*.

[2] Green J. (2014). The origins and evolution of fixed orthodontic appliances. *Dental Nursing*.

[3] Buonocore M. G. (1955). A simple method of increasing the adhesion of acrylic filling materials to enamel surfaces. *Journal of Dental Research*.

[4] Newman G. V., Snyder W. H., Wilson CE Jr, Hanesian D. (1965). Adhesives and orthodontic attachments. (preliminary investigation). *The Journal of the New Jersey State Dental Society*.

[5] Brown K. (2009). The impact of bonding material on bracket failure rate. *Vital*.

[6] Almosa N., Zafar H. (2018). Incidence of orthodontic brackets detachment during orthodontic treatment: a systematic review. *Pakistan journal of medical sciences*.

[7] Skidmore K. J., Brook K. J., Thomson W. M., Harding W. J. (2006). Factors influencing treatment time in orthodontic patients. *American Journal of Orthodontics and Dentofacial Orthopedics*.

[8] Stasinopoulos D., Papageorgiou S. N., Kirsch F., Daratsianos N., Jäger A., Bourauel C. (2018). Failure patterns of different bracket systems and their influence on treatment duration: a retrospective cohort study. *The Angle Orthodontist*.

[9] Bukhari O. M., Sohrabi K., Tavares M. (2016). Factors affecting patients' adherence to orthodontic appointments. *American Journal of Orthodontics and Dentofacial Orthopedics*.

[10] Barbosa I. V., Ladewig V. . M., Almeida-Pedrin R. R., Cardoso M. A., Santiago Junior J. F., Conti A. C. . C. F. (2018). The association between patient's compliance and age with the bonding failure of orthodontic brackets: a cross-sectional study. *Progress in Orthodontics*.

[11] Papageorgiou S. N., Pandis N. (2017). Clinical evidence on orthodontic bond failure and associated factors. *Orthodontic Applications of Biomaterials*.

[12] Sukhia R. H., Sukhia H. R., Azam S. I., Nuruddin R., Rizwan A., Jalal S. (2019). Prediction du taux de decollement des attaches en orthodontie : etude cohorte retrospective. *International Orthodontics*.

[13] Wang W. N., Yeh C. L., Fang B. D., Sun K. T., Arvystas M. G. (1994). Effect of H3PO4 concentration on bond strength. *The Angle Orthodontist*.

[14] Paschos E., Kurochkina N., Huth K. C., Hansson C. S., Rudzki-Janson I. (2009). Failure rate of brackets bonded with antimicrobial and fluoride-releasing, self-etching primer and the effect on prevention of enamel demineralization. *American Journal of Orthodontics and Dentofacial Orthopedics*.

[15] Sfondrini M. F., Cacciafesta V., Pistorio A., Sfondrini G. (2001). Effects of conventional and high-intensity light-curing on enamel shear bond strength of composite resin and resin-modified glass-ionomer. *American Journal of Orthodontics and Dentofacial Orthopedics*.

[16] Sardarian A., Malekpour B., Roshan A., Danaei S. M. (2019). Bleaching during orthodontic treatment and its effect on bracket bond strength. *Journal of Dental Research*.

[17] Naqvi Z. A., Shaikh S., Pasha Z. (2019). Evaluation of bond failure rate of orthodontic brackets bonded with Green Gloo-two way color changes adhesive: a clinical study. *Ethiopian Journal of Health Sciences*.

[18] Cobourne M. T., DiBiase A. T. (2015). *Handbook of Orthodontics E-Book*.

[19] Koupis N. S., Eliades T., Athanasiou A. E. (2008). Clinical evaluation of bracket bonding using two different polymerization sources. *The Angle Orthodontist*.

[20] Tang A. T., Björkman L., Isaksson L., Lindbäck K. F., Andlin-Sobocki A., Ekstrand J. (2000). Retrospective study of orthodontic bonding without liquid resin. *American Journal of Orthodontics and Dentofacial Orthopedics*.

[21] Roelofs T., Merkens N., Roelofs J., Bronkhorst E., Breuning H. (2017). A retrospective survey of the causes of bracket-and tube-bonding failures. *The Angle Orthodontist*.

[22] Ozer M., Bayram M., Dincyurek C., Tokalak F. (2014). Clinical bond failure rates of adhesive precoated self-ligating brackets using a self-etching primer. *The Angle Orthodontist*.

[23] Choo S. C., Ireland A., Sherriff M. (2001). An in vivo investigation into the use of resin-modified glass poly (alkenoate) cements as orthodontic bonding agents. *The European Journal of Orthodontics*.

[24] AL-Duliamy M. J. A. (2018). The effect of oral hygiene status on the bond failure rate of the orthodontic bracket. *Journal of Oral and Dental Research*.

[25] Cal-Neto J. P., Quintão C. A., de Oliveira Almeida M. A., Miguel J. A. M. (2009). Bond failure rates with a self-etching primer: a randomized controlled trial. *American Journal of Orthodontics and Dentofacial Orthopedics*.

[26] Millett D. T., Hallgren A., Cattanach D. (1998). A 5-year clinical review of bond failure with a light-cured resin adhesive. *The Angle Orthodontist*.

[27] Adolfsson U., Larsson E., Ogaard B. (2002). Bond failure of a no-mix adhesive during orthodontic treatment. *American Journal of Orthodontics and Dentofacial Orthopedics*.

[28] Linklater R. A., Gordon P. H. (2003). Bond failure patterns in vivo. *American Journal of Orthodontics and Dentofacial Orthopedics*.

[29] Elekdag-Turk S., Cakmak F., Isci D., Turk T. (2008). 12-month self-ligating bracket failure rate with a self-etching primer. *The Angle Orthodontist*.

[30] Mavropoulos A., Karamouzos A., Kolokithas G., Athanasiou A. E. (2003). In vivo evaluation of two new moisture-resistant orthodontic adhesive systems: a comparative clinical trial. *Journal of Orthodontics*.

[31] Menini A., Cozzani M., Sfondrini M. F., Scribante A., Cozzani P., Gandini P. (2014). A 15-month evaluation of bond failures of orthodontic brackets bonded with direct versus indirect bonding technique: a clinical trial. *Progress in Orthodontics*.

[32] Ahangar Atashi M. H., Shahamfar M. (2013). Long-term evaluation of clinical performance of direct-bonded brackets: an epidemiologic survey. *The Journal of Contemporary Dental Practice*.

[33] Kafle D., Mishra R. K., Hasan M. R., Saito T. (2020). A retrospective clinical audit of bracket failure among patients undergoing orthodontic therapy. *International journal of dentistry*.

